# Characterisation of an unusual cysteine pair in the Rieske carnitine monooxygenase CntA catalytic site

**DOI:** 10.1111/febs.16722

**Published:** 2023-01-19

**Authors:** Mussa Quareshy, Muralidharan Shanmugam, Alexander D. Cameron, Timothy D. H. Bugg, Yin Chen

**Affiliations:** ^1^ School of Life Sciences University of Warwick Coventry UK; ^2^ Department of Chemistry and Photon Science Institute The University of Manchester UK; ^3^ Department of Chemistry University of Warwick Coventry UK

**Keywords:** carnitine, cw‐EPR, cysteine residues, electron transfer, mononuclear Fe, Rieske monooxygenase

## Abstract

Rieske monooxygenases undertake complex catalysis integral to marine, terrestrial and human gut‐ecosystems. Group‐I to ‐IV Rieske monooxygenases accept aromatic substrates and have well‐characterised catalytic mechanisms. Nascent to our understanding are Group‐V members catalysing the oxidation/breakdown of quaternary ammonium substrates. Phylogenetic analysis of Group V highlights a cysteine residue‐pair adjacent to the mononuclear Fe active site with no established role. Following our elucidation of the carnitine monooxygenase CntA structure, we probed the function of the cysteine pair Cys206/Cys209. Utilising biochemical and biophysical techniques, we found the cysteine residues do not play a structural role nor influence the electron transfer pathway, but rather are used in a nonstoichiometric role to ensure the catalytic iron centre remains in an Fe(II) state.

## Graphical abstract

Carnitine monooxygenase (CntA) catabolises l‐carnitine into malic semialdehyde and trimethylamine (TMA), the latter being implicated in Fish odour syndrome. We characterised a pair of cysteine residues near the mononuclear‐Fe active site for which there was neither structural nor electron transfer pathway role but rather a nonstoichiometric reactivation of the catalytic mononuclear Fe (II) centre. This helps to better understand the role of these cysteines in quaternary amine catalysing Rieske monooxygenases.

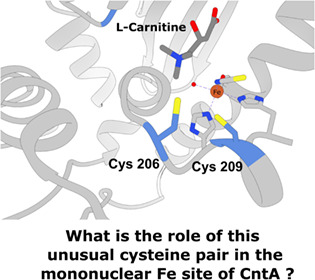

**Abbreviations**
CntA: carnitine monooxygenase
Cys: cysteine amino acid residue
DTNB: 5,5‐dithiobis‐(2‐nitrobenzoic acid)
cw‐EPR: continuous‐wave Electron Paramagnetic Resonance
TCEP: Tris(2‐carboxyethyl) phosphine

## Introduction

Rieske monooxygenases are comprised of five subgroups [1, 2]. Members of the subgroups I to IV, known to accept aromatic substrates [3, 4, 5, 6, 7], have been characterised extensively and their catalytic mechanisms elucidated [3, 8, 9, 10, 11]. Members of Group V have only been recently reported and associated with the oxidation/breakdown of the quaternary ammonium substrates such as glycine betaine [2], carnitine [1] and benzalkoniums [12]. In general, the underlying mechanism of function for Rieske monooxygenases is driven by an electron transfer from a reductase/ferredoxin partner protein facilitated through a series of [2Fe‐2S] clusters culminating at a mononuclear Fe centre [13] typically coordinated by a 2‐His‐1‐carboxylate (His‐His‐Asp) triad [14]. Substrates are oriented in the active‐site pocket, adjacent to the mononuclear Fe centre as well as a molecule of O_2_ [15] and the mechanism proceeds via an oxidative addition process [15].

**Fig. 1:**
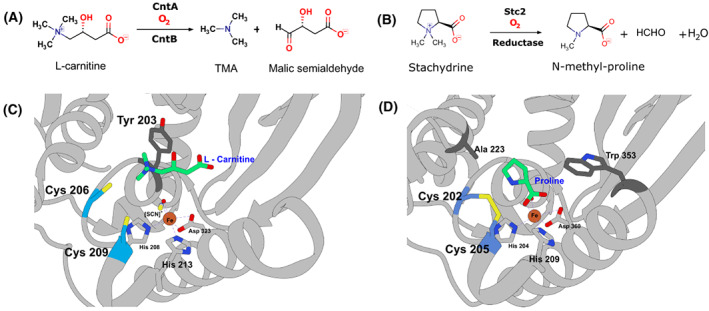
Comparisons of cysteine pairs in CntA and Stc2. (A) Overview of catalysis of carnitine monooxygenase CntA breaking down l‐carnitine into trimethylamine and malic semialdehyde. (B) Overview of catalysis of stachydrine demethylase, Stc2. (C) Cartoon representation of the CntA structure with carnitine substrate (Beige) coordinated by Tyr203 (Dark grey) and the Cys206 and Cys209 pair (Blue) in the mononuclear Fe centre active site shown as free thiols. (D) Cartoon representation of the Stc2 structure with the proline product (Beige) in the mononuclear Fe centre active site coordinated by Ala223 and Trp353 (Dark grey) and the Cys202 and Cys205 pair (Blue) are shown as a disulphide bridge. Figures generated in ucsf chimera 1.16 (Macintosh).

In all five groups of the Rieske oxygenase family, the coordinating residues of the [2Fe‐2S] and mononuclear Fe centres are highly conserved and are an identifying feature for this family [1, 16]. Unique to some Group V Rieske oxygenases is the presence of a cysteine pair Cys‐X‐X‐Cys adjacent to the catalytic mononuclear Fe site [17, 18] for which a role is yet to be reported. It is well‐understood that disulphide bridges between cysteine pairs are an integral structural feature of protein secondary and tertiary structures [19] crucial to stabilise and maintain folded protein architecture [20]. In the example of insulin, disulphide bridges can be established as interchain and intrachain linkages [21] and in some cases, even vicinal disulphide bonds are known [22, 23]. In the structure of cytochrome *bc*1, a disulphide bridge is observed in the vicinity of the [2Fe‐2S] Rieske cluster and was shown to be critical to stability and function of the cluster when the disulphide bridge is disrupted [24]. Analogous to the CntA and Stc2 enzymes, in terms of a Cys‐X‐X‐Cys motif, is the human thioredoxin TRx1 [25, 26], which can oxidise sulphurhydryls or reduce disulphide bonds on target proteins via its active site Cys32 and Cys35 residues. At present, there are reported structures for just two members of the group V clade; carnitine monooxygenase from *Acinetobacter baumannii* (CntA) [18] which catabolises carnitine to trimethylamine (TMA; Fig. 1A) and stachydrine demethylase (Stc2) [17] from *Sinorhizobium meliloti* (Fig. 1B). For the CntA structure, we reported the cysteine pair (Cys206‐Cys209) as free thiols (Fig. 1C) whilst the corresponding (Cys202‐Cys205) pair in Stc2 were assigned as a disulphide bridge (Fig. 1D). It should be noted that the Stc2 structure was obtained under anaerobic conditions but conversely aerobic conditions for CntA. Another distinguishing feature is that we observed the intact substrate in CntA; in Stc2, however, the substrate stachydrine has undergone two subsequent demethylation steps, which is observed as the end product.

In optimising our CntA protein purification methodology, we found the addition of TCEP [27] in purification and crystallography buffers was crucial. The addition of this phosphine‐reducing agent was key for long‐term stability [28] of purified fresh and frozen CntA. Our breakthrough in obtaining protein crystals was attributed to sodium thiocyanate (NaSCN) as an additive in the crystallography buffer, co‐bounding the mononuclear Fe centre. We noted that the presence of NaSCN at high concentrations impeded CntA activity. We also reported small‐molecule inhibitors for CntA with a crystal structure of the MMV12 inhibitor co‐bound to CntA under similar conditions where we still observe the cysteine pair as free thiols. These cysteines in CntA do not seem to coordinate the mononuclear Fe centre being ~ 5 Å away, nor do they interact with the carnitine or γ‐butyrobetaine substrates in any canonical manner, although the ammonium group of the substrate is oriented towards C206 and is ~ 4.4 Å away from the substrate. With almost no reported insight into this cysteine pair and no clear role attributed yet, in this work, we sought to focus our attention on the cysteine pair. We investigated with biochemical, spectroscopic and structural approaches to better understand these unusual residues proximal to the active site.

## Results and discussion

### Establishing the redox state of the Cys206 and Cys209 in CntA

**Fig. 2:**
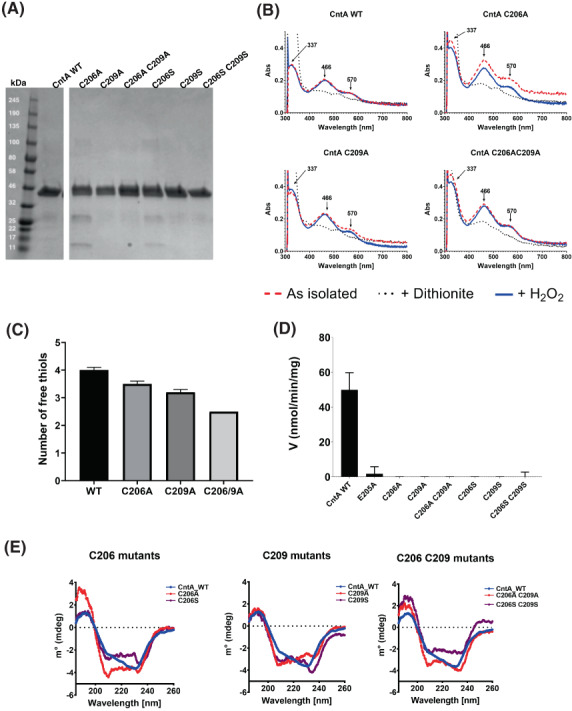
CntA mutagenesis and characterisation of mutants. (A) An SDS/PAGE electrophoresis gel of purified WT CntA and its mutants (~ 44 kDa). (B) As isolated UV–Vis spectra of WT and CntA mutants compared with UV–Vis spectra in the presence of dithionite or H_2_O_2_. (C) Quantification of free thiols with Ellman's reagent for WT and cysteine pair alanine mutants. (Error bars represent SD, *n* = 3). (D) Enzymatic activity comparison of mutants to WT showing a significant reduction in activity. (Error bars represent SD, *n* = 3). (E) Circular dichroism measurements of cysteine mutants relative to the WT (blue trace) in each subpanel.

In order to establish the roles of the Cys206‐Cys209 cysteine pair on the oxidation state of the purified CntA protein, we expressed and purified a series of alanine and serine (both single and double) mutants for the C206 and C209 residues (Fig. 2A). Using UV–Vis spectroscopy, we studied the as‐isolated proteins in the presence of excess (2 mm) sodium dithionite and hydrogen peroxide and observed no differences to that of the WT (Fig. 2B). We sought to quantify the number of free thiol groups in the WT CntA and the mutants using Ellman's reagent [(5,5‐dithiobis‐(2‐nitrobenzoic acid), DTNB)], expecting five solvent accessible cysteine residues in the WT: Cys12, Cys125, Cys160, Cys206 and Cys209. We only observed four free thiols in the WT, however (Fig. 2C). Given that the C206 and C209 residues are relatively close to one another (3.8 Å), both are proximal to the Fe centre, it is plausible that only one of these cysteines can bind the 2‐nitro‐5‐thiobenzoate (TNB) at once. However, we observed between 2 and 3 free thiols in the C206A/C209A double mutants and between 3 and 4 in the single mutants. Overall, this result corroborates with the observation from CntA structure and suggests that the cysteines C206 and C209 do not appear to form a disulphide bridge in the WT protein.

### Cys206 and Cys209 are crucial for CntA enzyme activity but the C209A mutation does not affect the overall protein structure

When assayed for activity, we observed a significant reduction in activity for C206A, C209A, C206S and C209S mutants (Fig. 2D). We studied the secondary structure of these variants with Circular Dichroism measurements and observed the most pronounced difference between the WT and C206A mutant, with the overall secondary structure also different for the other CntA mutational variants (Fig. 2E). Secondary structure estimation [29, 30] (Table S1) of the CD data shows variations of secondary structure in all C206 and C209 mutants relative to WT. We have also included quantification for a previously reported E205A, which showed an identical CD spectra to the WT with small secondary structure differences [1, 18]. We looked at the stability of these mutants with a thermal shift assay (TSA) with C206A producing a 1.42 °C decrease in melting temperature, indicating a slight destabilisation of the protein relative to WT CntA (Table S2), agreeing with the CD data and indicating some instability. Overall, we saw differences in the CD and melting points between corresponding Alanine and Serine mutations of C206 and C209 single mutants with no obvious pattern or uniform trends in instability. We conclude that within these mutations there is some, albeit small influence of the C206 and C209 residues on the secondary structure of CntA and that overall, the proteins were folded correctly.

**Fig. 3:**
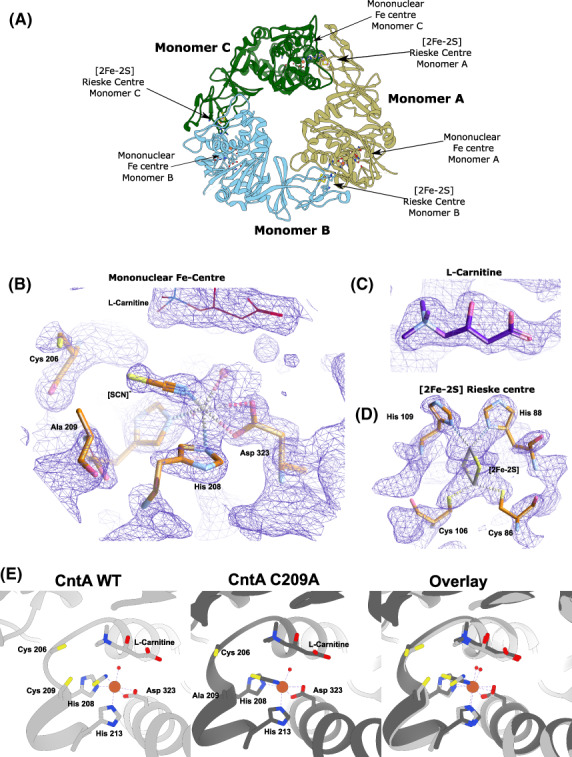
Crystal structure of the CntA C209A mutant. (A) Overview of CntA 209A Trimeric structure with head‐to‐tail orientation of [2Fe‐2S] Rieske centre and Mononuclear Fe Centre. (B) A 2mF_o_ − DF_c_ map (Blue) at 1.0σ of the mononuclear Fe centre with coordinated [SCN]^−^ co‐factor as well as C206 and C209A residues. (C) A 2mF_o_ − DF_c_ map (Blue) at 1.5σ of carnitine substrate. (D) A 2mF_o_ − DF_c_ map (Blue) at 2.0σ of the [2Fe‐2S] Rieske centre. (E) A cartoon representation of the CntA WT active site (Light Grey) and the C209A structure (Dark Grey) separately and overlaid, respectively, to show the substrate and mononuclear Fe coordination are unchanged whilst the orientation of the [SCN]^−^ and C206 are tilted differently in the C209A mutant but still [SCN]^−^ is coordinated to the Fe centre. Figure generated in ucsf chimera 1.16 (Macintosh).

To better understand the structural basis of these cysteines on CntA enzyme activity, we set out to solve their structures but only managed to successfully obtain the structure of the C209A mutant with carnitine co‐bound which was refined at a resolution of 1.8 Å (PDB 6Y9C, Table S3). The CntA 209A + carnitine structure is a α_3_ homotrimer (Fig. 3A), with the mononuclear Fe centre coordinated by a 2‐His‐1‐Asp triad, a water molecule and a [SCN]^−^ co‐factor from the protein crystallisation buffer additive sodium thiocyanate (Fig. 3B) analogous to the WT structure. The carnitine substrate was observed as expected in the electron density (Fig. 3C) and in the [2Fe‐2S] Rieske centre (Fig. 3D). The carnitine substrate sits above the mononuclear Fe centre in a similar position relative to the WT (Fig. 3E). The orientation of the [SCN]^−^ co‐factor is tilted differently in the CntA C209A structure vs. the WT where the sulfur of the [SCN]^−^ occupies the region where we would expect the thiol group of C209A to be. Overall, the structures of C209A and WT CntA are very similar with a rmsd of 0.461 Å (Fig. 3E), suggesting the mutation of C209A had minimal impact on the overall structure of CntA which contrasts the observations CD and TSA assays. We acknowledge that in the C209A structure represents a snapshot of the protein structure and that the presence of l‐carnitine as well the [SCN]^−^ [31] may have promoted increased protein stability.

### Cys206 and Cys209 mutants do not impede electron transfer into the catalytic Fe Centre

**Fig. 4:**
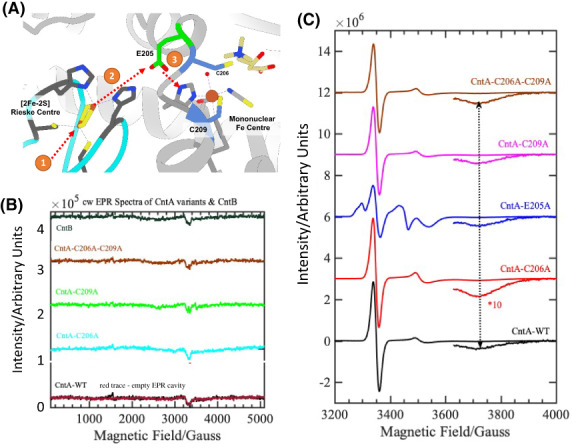
Electronic Paramagnetic Resonance (EPR) measurements of CntA cysteine mutants relative to CntA WT. (A) A cartoon representation of the interface of two monomers of CntA (Cyan and Light Grey). The electron transfer pathway is shown as (1) Electron transfer into the [2Fe‐2S] Rieske centre (Dark Grey residues) of CntA, (2) onto the E205 bridging residue (green) and followed by (3) transfer into the Mononuclear Fe centre. The Cys 206 and Cys 209 pair (Blue) are shown to be adjacent to the E205 bridge and mononuclear Fe centre and investigated for their role in this transfer pathway. Figure generated in ucsf chimera 1.16 (Macintosh). (B) Continuous‐wave (cw)‐EPR spectra of AbCntA‐WT/AbCntB and its variants measured as a frozen solution at 20 K. The EPR spectra of AbCntA‐WT (black trace), AbCntA‐C206A (cyan trace), AbC209A (green trace), AbC206AC209A (brown trace) and AbCntB (dark green trace) show weak EPR signals at ~ 3300 G, which are identical to that of the empty EPR cavity (background signal; red trace) overlaid on to the AbCntA‐WT spectrum. This implies that mutation has no effect on the redox state of the Rieske and mononuclear Fe centre of the AbCntA enzyme. Conditions—microwave power 30 dB (0.2 mW), modulation amplitude 5 G, temperature 20 K; time constant 82 ms, conversion time of 12 ms, sweep time of 120 s, the receiver gain set to 30 dB and an average microwave frequency of 9.383 GHz. C: The narrow‐swept, cw‐EPR spectra of AbCntA‐WT and its cysteine variants in the presence of AbCntB + NADH + Carnitine show the observation of identical EPR characteristics suggest that the electron transfer pathway might be similar in AbCntA‐WT (black trace) and also in the single/double mutants, AbCntA‐C206A (red trace), AbCntA‐C209A (magenta trace) and AbCntA‐C206AC209A (brown trace); the high‐field EPR transition observed at ~ 3720 G is barely visible from the baseline of the EPR spectra—to make it visible to the naked eye, the high‐field EPR signals are zoomed‐in as indicated by the dotted double‐headed black arrow; this comparison clearly shows that mutation has no effect on the electron transfer pathway(s); however, the mutation of the bridging carboxylate, E205A (blue trace) between mononuclear iron and Rieske centre has affected the efficient electron transfer pathways; conditions—as in Fig. 4B.

To understand the inactivity and/or significantly reduced catalytic activity of the single and double mutants, C206A, C209A and C206AC209A, cw‐EPR spectra were measured on these mutants and qualitatively compared with that of the CntA‐WT enzyme. We previously characterised the complete electron transfer pathway in the CntA WT protein in the presence of a reductase, CntB + NADH and carnitine as the substrate [18]. We demonstrated the E205 bridging residue plays an integral role in the electron transfer pathway (Fig. 4A), with the E205A enzyme variant showing significantly reduced catalytic activity explained by a retardation of the electron transfer process from the reduced, [2Fe‐2S]^1+^ clusters of CntB (ferredoxin)/CntA(Rieske) centre into the mononuclear Fe centre. Should the cysteine pair also be part of the electron transfer pathway, we would expect to observe the same retardation. First, near‐identical EPR spectra are observed for the resting state when CntA‐WT and single mutant, CntA‐C209A were purified without TCEP buffer, implying that TCEP has no direct role in keeping the catalytic Fe centre in its ferrous oxidation state (Fig. S1). We then measured the cw‐EPR spectra of the purified single (C206A and C209A) and double (C206AC209A) mutants of CntA protein at 20 K and observed an EPR silent state similar to that of the WT protein [18] (Fig. 4B). It is consistent with the oxidised [2Fe‐2S]^2+^ Rieske centre and a catalytic, mononuclear iron centre in its ferrous state, demonstrating these mutations have no effect on the redox properties of the [2Fe‐2S]^2+^ and mononuclear iron centres in CntA enzyme. When the mutants were measured in the presence of CntB + NADH + Carnitine, the spectra show identical EPR traces to that of the WT (Fig. 4C). This implies that none of the C206/C209 single and double mutants impeded the electron transfer pathway, in contrast to what has been observed for the E205A mutant (Fig. 4C; blue trace). Together, these data suggest that these cysteines are unlikely to be part of the normal electron transfer pathway. The additional EPR signals observed around 3250–3300 G and 3400–3600 G in the blue trace are due to the one‐electron reduced, ferredoxin [2Fe‐2S]^+1^ due to inefficient electron transfer as we reported previously [18]. The broadening observed at 3550 G is due to the overlapping of reduced, ferredoxin/Rieske and activated mononuclear Fe EPR signals at this field position.

### Cys206 and Cys209 do not affect peroxide shunt mechanism

**Fig. 5:**
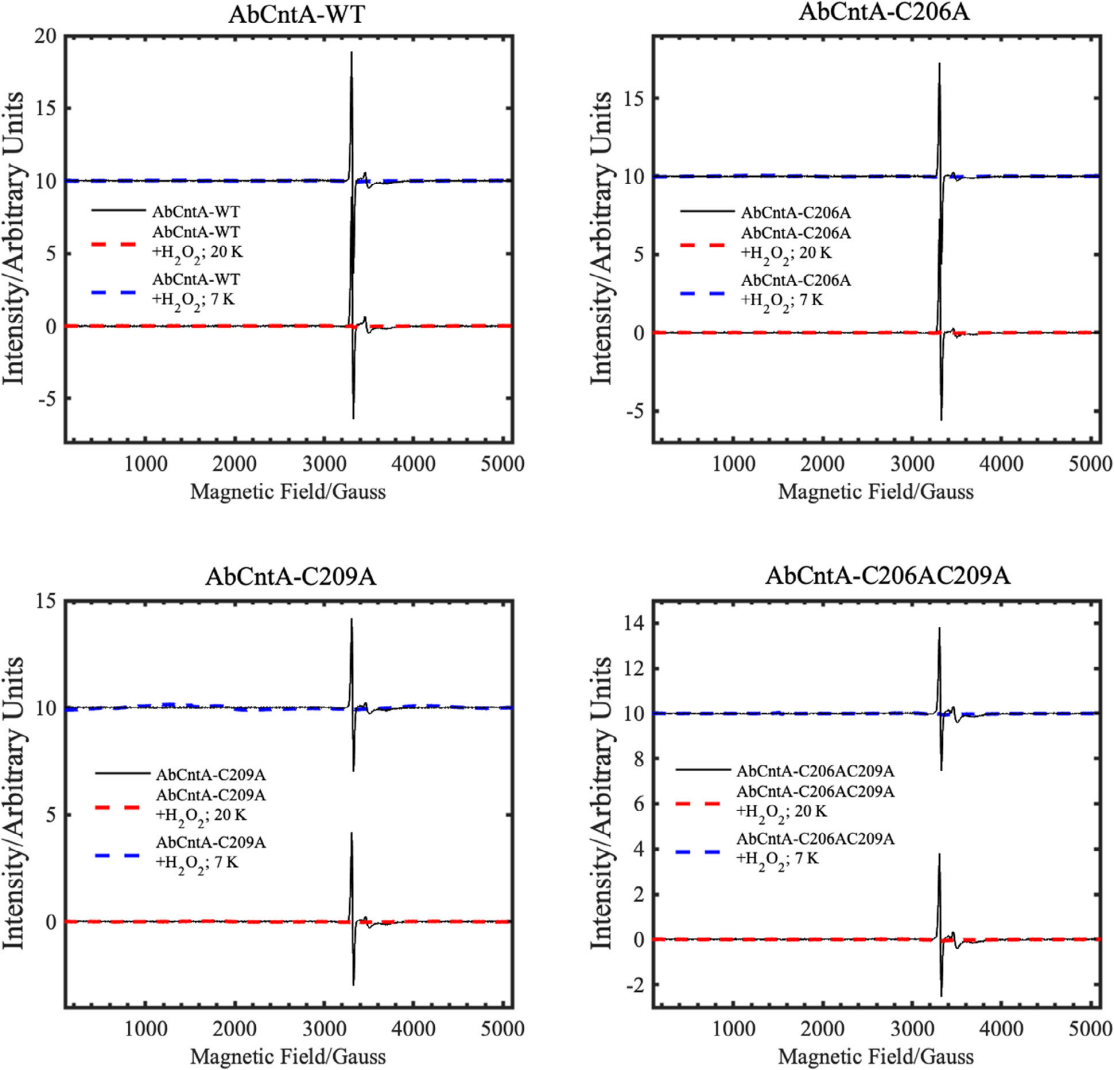
cw‐EPR spectra of the ‘as‐isolated’ AbCntA‐WT + H_2_O_2_ and its mutants + H_2_O_2_ in the absence of substrate, carnitine. The spectra measured at both 20 K (red traces) and 7 K (blue traces) are overlaid on the AbCntA‐WT + AbCntB + NADH + carnitine (black traces) to show they are EPR silent and no formation of the high‐spin, ferric EPR signals at low magnetic field. Conditions as described in Fig. 4B.

**Fig. 6:**
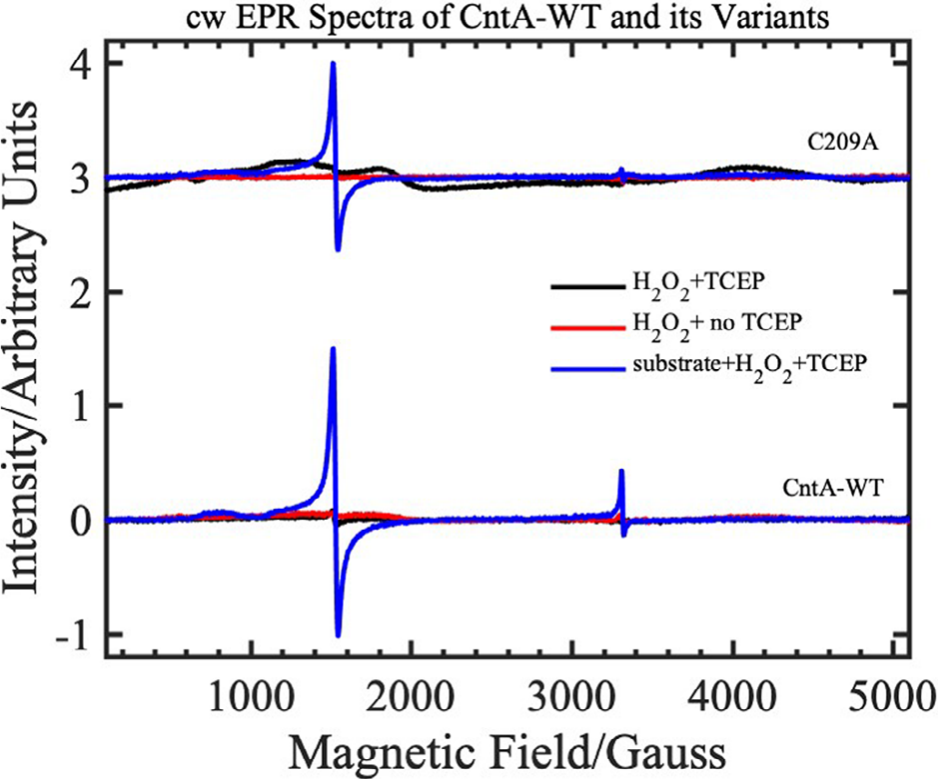
cw‐EPR spectra of the ‘as‐isolated’ AbCntA‐WT + H_2_O_2_ and its single mutant, AbCntA‐C209A+ H_2_O_2_ in the presence of substrate, carnitine. The purified oxidase domain, AbCntA (WT + H_2_O_2_ and C209A‐mutant + H_2_O_2_) with (black traces) and without (red traces) TCEP shows identical EPR spectra (EPR silent state) when measured in the absence of substrate, but intense, high‐spin, ferric EPR signals are observed at low magnetic field in the presence of carnitine (blue traces). A small signal (*g* = 2) of unknown origin is also observed at ~ 3350 G, as observed in other Rieske nonheme enzymes [32]. Conditions as described in Fig. 4B.

Like P450 oxygenases, CntA can also perform a peroxide shunt oxidation of the substrate using H_2_O_2_ in the absence of NADH and the reductase CntB [18]. To determine whether C206/C209 plays a role in the H_2_O_2_‐mediated peroxide shunt mechanism, EPR spectra were measured on the *Ab*CntA‐WT, single (*Ab*CntA‐C206A and *Ab*CntA‐C209A) and double (*Ab*CntA‐C206AC209A) mutants (Figs 5 and 6). In the absence of the substrate (Fig. 5), these spectra show no indication of high‐spin, ferric EPR signals at low magnetic field, between 0 and 2000 G in the presence of an oxidising agent (4 mm H_2_O_2_) at 20 K (red dotted traces) and 7 K (blue dotted traces), respectively. For a comparison purpose, all spectra were overlaid on to the EPR spectrum of the *Ab*CntA‐WT + *Ab*CntB + NADH + carnitine (black traces in all four panels; Fig. 5). No differences between the WT, single mutants or the double mutant were observed. In the presence of carnitine, however, the spectra (Fig. 6) show intense, high‐spin, ferric EPR signals at ~ 1500 G (blue traces) when CntA‐WT and *Ab*CntA‐C209A were treated with H_2_O_2_. This implies that *Ab*CntA‐C209A mutation does not impair the peroxide‐shunt mechanism.

### Cys206 and Cys209 may play a role in reactivation of the mononuclear Fe Centre

**Fig. 7:**
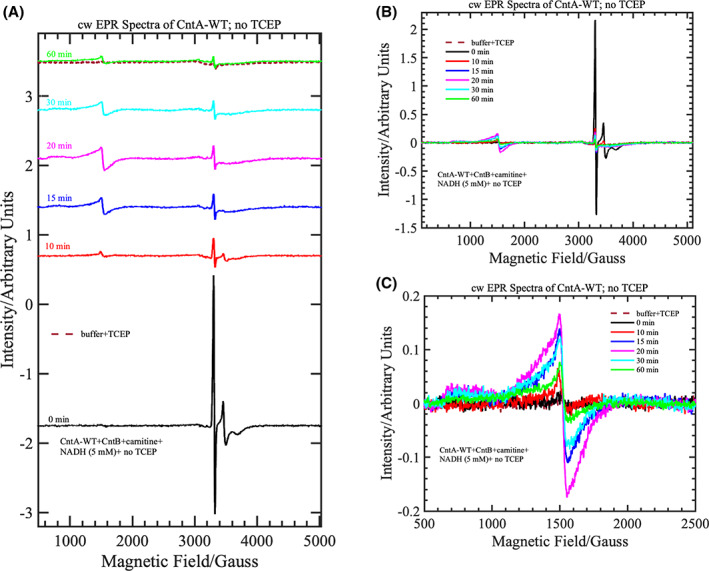
Annealing studies performed on the AbCntA‐WT enzyme in the absence of TCEP buffer at room temperature. (A) cw‐EPR spectra of AbCntA‐WT + AbCntB + NADH (5 mm) + carnitine in the absence of TCEP in the buffer, measured as a frozen solution at 20 K. This sample was annealed/thawed at room temperature for the specified duration mentioned in the figure legend and measured again at 20 K. The observed changes in the spectrum are monitored as a function of annealing (Fig. 8) time. The spectra in panel (A) are overlaid in panel (B) to monitor the changes in intensity of the high‐spin (*S* = 5/2)/low‐spin (*S* = ½), ferric EPR signals when the samples were annealed at RT; The EPR signal observed at ~ 1500 G is zoomed‐in on panel (C) to monitor the high‐spin, ferric EPR signal of the mononuclear Fe centre. The dotted wine‐red traces overlaid on panels (A and C) are EPR spectra of TCEP in the buffer (negative control)‐show that the EPR signals observed around ~ 3300 G are likely arising from the background/buffer. Conditions as described in Fig. 4B.

**Fig. 8:**
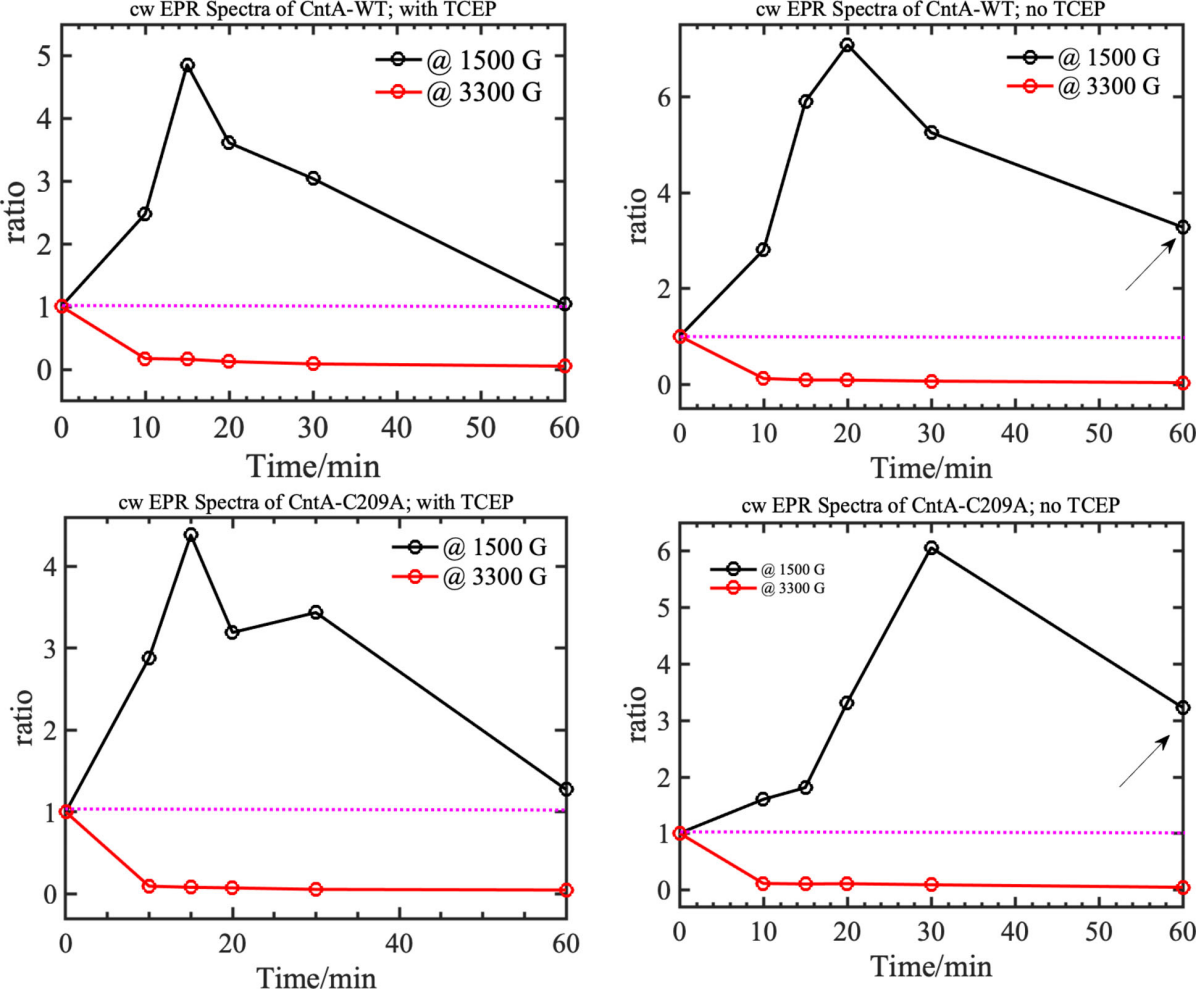
Monitoring the change in intensity of the high‐spin/low‐spin EPR signals of the AbCntA‐WT and AbCntA‐C209A mutant after annealing the samples at room temperature. Normalised (the plotted EPR signals in each spectrum is normalised to the maximum of the signals from the EPR spectrum before annealing of the sample; zero time) Intensity of the EPR signals at ~ 3300 G (see Fig. 7B)/~ 1500 G (see Fig. 7C) has been monitored as a function of annealing for AbCntA‐WT + AbCntB + NADH (5 mm) + carnitine and AbCntA‐C209A + AbCntB + NADH + carnitine in the absence (top‐right and bottom‐right) and presence (top‐left and bottom‐left) of TCEP in the buffer. Please refer to Fig. 7B,C for the EPR signals at ~ 1500 G and 3300 G, respectively. The black arrows on top‐right and bottom‐right panels show that the oxidised, ferric signal is not completely decayed/reduced to EPR silent or catalytically active ferrous centre. Conditions as described in Fig. 4B.

The enzymology, structure and EPR results discussed thus far do not support a role of these cysteines in electron transfer to the mononuclear Fe centre in CntA catalysis, nor for their involvement in maintaining the structure of CntA. Another hypothesis is that the reduced cysteines in CntA may be needed to reactivate the mononuclear Fe(II) centre when it is occasionally oxidised to the inactive Fe(III) centre. A similar phenomenon has been observed for prolyl hydroxylase, a 2‐oxoglutarate‐dependent dioxygenase that also has a mononuclear iron (II) centre, which requires the reductant, ascorbate in order to keep the iron centre in the ferrous state during catalysis [33, 34, 35]. In the absence of ascorbate, the enzyme can only carry out limited rounds of turnovers due to the oxidation of the enzyme‐bound iron (II) to iron (III) caused by the so‐called uncoupled reaction of decarboxylation of 2‐oxoglutarate [33, 34, 35]. To investigate the involvement of C206/C209 and the reduced thiol groups in CntA catalysis, we performed annealing of the [CntA‐WT + CntB + NADH + carnitine] and [CntA‐C209A + CntB + NADH + carnitine] samples prepared in the presence or absence of TCEP in the buffer. The EPR spectra of the [CntA‐WT + CntB + NADH + carnitine] and [CntA‐C209A + CntB + NADH + carnitine] samples purified in the presence of TCEP and flash‐frozen immediately after the addition of all four components show no evidence of high‐spin, ferric EPR signals at low magnetic fields (Fig. 7). When the [CntA‐WT + CntB + NADH + carnitine] sample was annealed at room temperature for the specified duration mentioned in the figure caption, development of the high‐spin, ferric EPR signals (at ~ 1500 G) was observed, which reached a maximum at around 15 min of annealing at room temperature (Fig. 7B,C). A similar trend was observed for the [CntA‐C209A + CntB + NADH + carnitine] single mutant sample. Further annealing of the samples led to the slow decay/disappearance of the signal as demonstrated by the ratio of the intensity of the high‐spin, ferric EPR signal at ~ 1500 G before and after annealing as a function of annealing time (Fig. 8; top‐left and bottom‐left). Identical experiments performed on the same samples purified in the absence of TCEP show a slightly different annealing trend, for example the maximum in the high‐spin, ferric EPR signal was observed ~ between 20 and 30 min for the CntA‐WT and CntA‐C209A samples. Interestingly, when the samples were annealed for a longer duration, the high‐spin, ferric EPR signals did not completely disappear (Fig. 8; top‐right and bottom‐right). These two comparisons clearly demonstrate that the presence of TCEP is critical for both mutant and wild‐type proteins. The TCEP may either directly assist the reduction in the inactive Fe(III) centre to the catalytically active, Fe(II) or could keep the cysteine in the reduced thiol state, which could subsequently reduce the Fe(III) to Fe(II).

**Fig. 9:**
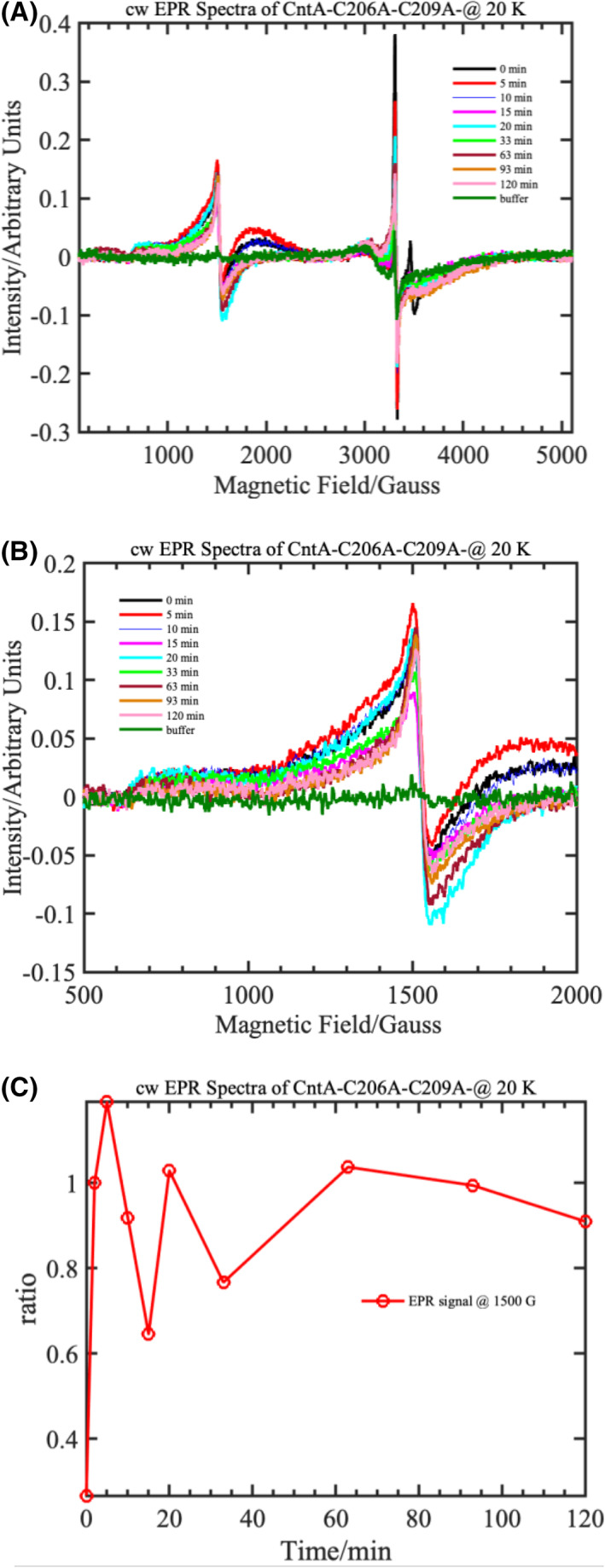
Annealing studies performed on the double mutant, AbCntA‐C206A‐C209A enzyme in the presence of TCEP buffer at room temperature.(A) cw‐EPR spectra of AbCntA‐C206AC209A + AbCntB + NADH (5 mm) + carnitine in the presence of TCEP in the buffer, measured as a frozen solution at 20 K. This sample was annealed/thawed at room temperature for the specified duration mentioned in the figure legend and measured again at 20 K. The overlaid spectra in panel (A) shows the overall changes in intensity of the high‐spin (*S* = 5/2)/low‐spin (*S* = ½), ferric EPR signals when the sample was annealed at RT; the observed changes in the spectrum are monitored as a function of annealing (Panel B) by plotting the normalised (the signals at ~ 1500 G is normalised to the maximum of the signals from EPR spectrum before annealing the sample; zero time) intensity of the EPR signal at ~ 1500 G (Panel C). The EPR signal observed at ~ 1500 G is zoomed‐in on panel (B) to monitor the high‐spin, ferric EPR signal of the mononuclear Fe centre. This plot shows that the majority of the ferric, high‐spin centre remains ‘unaffected/oxidised’ even when TCEP is present in the buffer. Conditions as described in Fig. 4B.

To probe this further, annealing experiments were performed on the CntA‐C206A‐C209A double mutant purified in the presence of TCEP buffer. When the sample was annealed at room temperature, high‐spin, ferric EPR signals were observed at ~ 1500 G (Fig. 9A,B) and the intensity of this signal oscillates for the first 30 min of annealing but does not completely disappear (Fig. 9C). Further annealing showed that the signal intensity remained constant within experimental error, and no decay of the signals was observed with up to 2 h of annealing at room temperature. If TCEP were responsible or directly involved in the reduction in Fe(III) into catalytically active Fe(II) centre, then we would have observed the disappearance of the high‐spin, Fe(III) EPR signals. The absence of this behaviour implies that cysteine residues are necessary to keep the catalytic Fe centre in the reduced state when ‘off‐pathway oxidation’ occurs at the catalytic centre. To make this point clear, the annealed EPR spectra of [CntA‐WT + CntB + NADH + carnitine] and [CntA‐C206A‐C209A + CntB + NADH + carnitine] in the presence of TCEP are compared in Fig. S2. It is clear from the comparison that the ferric EPR signals have been rescued in the [CntA‐WT + CntB + NADH + carnitine] (Fig. S2A; green trace – 60 min), whereas this rescue process is absent/lacked in the [CntA‐C206A‐C209A + CntB + NADH+ carnitine] double mutant (Fig. S2B; pink trace – 120 min) in the presence of TCEP. The ‘ratio vs. time’ plots confirms that the oxidised, Fe(III) centre cannot be rescued (Fig. S2C,D) back to its catalytically active form in the double mutant sample. It is clear that TCEP is required to keep the cysteine in the reduced thiol state, which in turn protects the catalytic Fe(II) centre in the ferrous state when ‘off‐pathway oxidation’ occurs.

## Conclusions

In elucidating a crystal structure of CntA [18], we were afforded an insight into how quaternary amine substrates are perceived in the active site. A unique feature observed from the CntA structures is the presence of two reduced Cys in the close vicinity of the active centre. Appearance of such Cys near the catalytic mononuclear iron centre is rare in Rieske oxygenases. While in the homologous Stc2, the Cysteines form a disulphide [17], the data we collected in this study do not appear to support a structural role of the Cysteines in CntA. First, these two Cys residues were present in the reduced state in the CntA crystal structure (Fig. 1) consistent with an analysis of reduced thiols in CntA using Ellman's Reagent (Fig. 2). Second, the crystal structure of the CntA C209A mutant showed no obvious impact to the substrate binding nor to the overall active site (Fig. 3D). Admittedly, the mutation of C206/C209 does appear to exert some impact on the secondary structure of the enzyme in CD analysis (Fig. 2E). These Cys residues do not appear to be involved in the normal electron transfer pathway from the reduced Rieske centre to the catalytic mononuclear iron centre, since their EPR spectra were largely undistinguishable from that of the WT (Fig. 4). This is in sharp contrast to the spectra of the E205A mutant, which displayed retarded electron transfer from the Rieske centre to the catalytic mononuclear Fe centre (Fig. 4). Furthermore, using H_2_O_2_ as the electron donor to bypass the need for NADH and the reductase CntB, we showed that these Cys residues do not appear to be required for the substrate‐dependent activation of the mononuclear Fe centre and the formation of the high‐spin S = 5/2 species (Figs 5 and 6), a mechanism that is consistent with the peroxide shunt. We therefore postulate that these Cys residues are likely to be involved in maintaining Fe(II) in its reduced state in catalysis. Data from EPR experiments shown in Figs 7, 8, 9 appear to suggest that these Cys residues are indeed involved in nonstoichiometric reactivation of the catalytic Fe (II) centre. Occasional oxidation of Fe(II) to Fe(III) is a problem encountered in other nonheme iron‐dependent oxygenases, which in prolyl hydroxylase is solved by nonstoichiometric reduction by ascorbate [35], and in *Pseudomonas putida* catechol 2,3‐dioxygenase by the use of a dedicated [2Fe‐2S] ferredoxin [36]. To the best of our knowledge, the use of two active site Cys residues to achieve this role is a novel mechanism to solve this problem. EPR experiments, however, only provided a static view of unpaired electrons at a given time. As such, the data presented here using EPR cannot provide a resolution of Fe on the single turnover timescale and the impact of Cys mutations on the EPR‐active Rieske centre and the catalytic Fe centre. Clearly future experiments on single turnover enzyme kinetics will provide further insight into the intriguing role of these unique Cysteine residues in CntA catalysis. It also remains to be seen whether such a mechanism operates in other group V Rieske oxygenases, where a homologous Cys pair appears common.

## Materials and methods

Protein expression, purification, UV–visible enzyme assays and biochemical enzyme assays work were performed as reported previously [18].

### UV–visible spectra characterisation

CntA WT and mutant protein material were freshly purified as reported previously [18] and prepared as 4 mg·mL^−1^ solutions in final volume of 160 μL, referred to ‘as isolated’. For each protein (WT and mutant), additional samples were prepared: (a) ‘+Dithionite’ with excess Dithionite reducing agent added at 2 mm final concentration and (b) ‘+H_2_O_2_’ hydrogen peroxide oxidising agent added at 2 mm final concentration. Samples were loaded onto a 96‐Well UV Transparent Plate (Thermo Fisher Scientific, Loughborough, UK, catalogue no. 8404) and read on a BMG FLOUstar Omega 96‐well plate reader scanning between wavelengths 300 and 800 nm recording absorbance and sampling in 1 nm intervals.

### Circular dichroism

All proteins were buffer exchanged into a pH 7.0, 0.2 m sodium phosphate buffer with 0.01 m NaCl on a PD‐10 column and concentrated to 0.1 mg·mL^−1^ final concentration. Using a 0.1 mm path length quartz cuvette, the samples were analysed on a JASCO J‐1500 at 20 °C and data were collected between 260 and 180 nm with 8 scans per sample. Secondary structure estimation was performed using the bestsel server [29, 30].

### Thermal shift assay

The assay was adapted from reported protocols [37, 38]. The CntA WT and mutant proteins were assayed at 2.5 μm with 1× SYPRO orange on a Bio‐Rad CFX Connect™ Real‐Time PCR instrument in a sample volume of 50 μL in triplicates for each sample. An initial 3‐min equilibration at 20 °C followed by 0.5 °C increments to 90 °C every 30 s recording the FRET signal at each stage. The data were auto‐processed in the bio‐rad software (Watford, UK) from which melting temperature was obtained and reported.

### EPR spectroscopy

All EPR samples were prepared in a 10 mm HPEPS buffer with 250 mm NaCl, 0.5 mm TCEP and 10% glycerol (v/v) (pH 7.6) in an aerobic condition. Samples containing ~ 200 μm *Ab*CntA, 75 mm nicotinamide adenine dinucleotide (NADH) were transferred into 4‐mm Suprasil quartz EPR tubes (Wilmad LabGlass, Vineland, NJ, USA) and frozen in liquid N_2_. Annealing of the samples was performed at room temperature for the specified time‐duration in the figure caption. All EPR samples were measured on a Bruker EMX‐Plus EPR spectrometer (Coventry, UK) equipped with a Bruker ER 4112SHQ X‐band resonator as reported previously [39]. Sample cooling was achieved using a Bruker Stinger [40] cryogen‐free system mated to an Oxford Instruments ESR900 cryostat, and temperature was controlled using an Oxford Instruments MercuryITC (Abingdon, UK). The optimum conditions used for recording the spectra are given below; microwave power 30 dB (0.2 mW), modulation amplitude 5 G, time constant 82 ms, conversion time 12 ms, sweep time 120 s, receiver gain 30 dB and an average microwave frequency of 9.383 GHz, temperature 20 K. cw‐EPR spectra for the AbCntA‐WT, single (C206A and C209A) and double (C206AC209A) mutants of CntA were recorded in the presence and absence of CntB + NADH + carnitine, as reported previously [18, 41].

### Protein crystallography

Protein crystallography was performed as previously reported. In brief, CntA C209A protein crystals were prepared at 7.5 mg·mL^−1^ in conditions of 18% (w/v) PEG 3350, 10 mm NaCl and 0.5 mm TCEP with substrates present at 1 mm. Red hexagonal crystals developed at 22 °C between 24 and 48 h. Crystals were cryoprotected in an equivalent solution to the mother liquor supplemented with 5% glycerol and flash cooled in liquid nitrogen.

The crystals were mounted robotically on the i04 beamline (CntA C209A mutant) at the Diamond Light Source (Harwell Science and Innovation Campus, Didcot, UK). Due to the presence of ice‐rings and anisotropy, the data were not ideal, and we truncated the data set with a resolution cut‐off at 1.8 Å. The selected images were processed with the Dials [42] GUI in ccp4i2 [43, 44] performing Indexing, refinement and integration steps, followed by scaling with Aimless [45]. We used the CntA + Carnitine structure (PDB code: 6Y8S) as a model for molecular replacement in phaser [46]. Autobuilding in phenix.autobuild was followed by iterative rounds of manual building in coot [47] interspersed with refinement in phenix [48].

### Free thiol quantification using the Ellman's reagent (DTNB)

A standard calibration range of a twofold dilution series between 3.2 and 0.1 mm of l‐cysteine hydrochloride (Sigma Aldrich, Gillingham, UK) were made. All protein samples were buffer exchanged into a 100 mm sodium phosphate buffer pH 8.0 (RB) and adjusted to a concentration between 5 and 6 mg·mL^−1^. A stock concentration of Ellman's reagent (DTNB) was made at 4 mg·mL^−1^. Two hundred and fifty microlitres of standard and unknowns, respectively, was added to 50 μL of the DTNB stock and 2.5 mL of RB and left for 15 min. Two hundred microlitres aliquots of this mix was assayed in triplicate in a 96‐well plate in a BMG FLOUstar Omega 96‐well plate reader measuring the absorbance at 412 nm. The absorbance values for the unknown samples were extrapolated from the standard curve, and the resulting molar concentration was divided by the molar concentration of protein used to yield the number of free thiol groups.

### Chemical structures and protein structure depictions

Marvin was used for drawing, displaying and characterising chemical structures, substructures and reactions, marvin v19.10.0, 2019, chemaxon (http://www.chemaxon.com). Molecular graphics and analyses were performed with ucsf chimaera [49].

## Conflict of interest

The authors declare no conflict of interest.

## Author contributions

MQ, MS and YC designed research. MQ carried out the protein expressions, protein purifications, enzyme assays, data analysis, protein crystal preparation and EPR sample preparation. MS carried out all EPR experiment measurements and analysis. MQ and ADC collected crystallographic data, processed, solved and refined the structure. MQ, MS and YC wrote the manuscript with contributions from ADC and TDHB. YC, ADC and TDHB sourced funding and formulated the original research idea.

### Peer review

The peer review history for this article is available at https://publons.com/publon/10.1111/febs.16722.

## Supporting information


**Fig. S1.** cw‐EPR spectra of CntA WT and C209A mutant with and without TCEP.
**Fig. S2.** Key figures from main manuscript to illustrate off‐pathway oxidation.
**Table S1.** Estimated CD secondary structure content.
**Table S2.** Thermal Shift assay summary of temperature shifts relative to CntA WT.
**Table S3.** Crystallographic statistics.

## Data Availability

The CntA C209A + Carnitine structure is deposited in the PDB repository with accension code PDB Code: 6Y9C.
