# Supplementary material for: Characterisation of an unusual cysteine pair in the Rieske carnitine monooxygenase CntA catalytic site
Source: FEBS J. 2023 Jan 19;290(11):2939–53. doi: 10.1111/febs.16722 (PMC10952381; doi:10.1111/febs.16722)
Supplement: Supplementary file 1 — Fig. S1. cw‐EPR spectra of CntA WT and C209A mutant with and without TCEP. Fig. S2. Key figures from main manuscript to illustrate off‐pathway oxidation. Table S1. Estimated CD secondary structure content. Table S2. Thermal Shift assay summary of temperature shifts relative to CntA WT. Table S3. Crystallographic statistics. [file FEBS-290-2939-s001.pdf]

Characterisation of an unusual cysteine pair in the Rieske carnitine monooxygenase CntA catalytic site

Mussa Quareshy, Muralidharan Shanmugam, Alexander D Cameron, Timothy D.H. Bugg, Yin Chen

Supplementary figures and tables

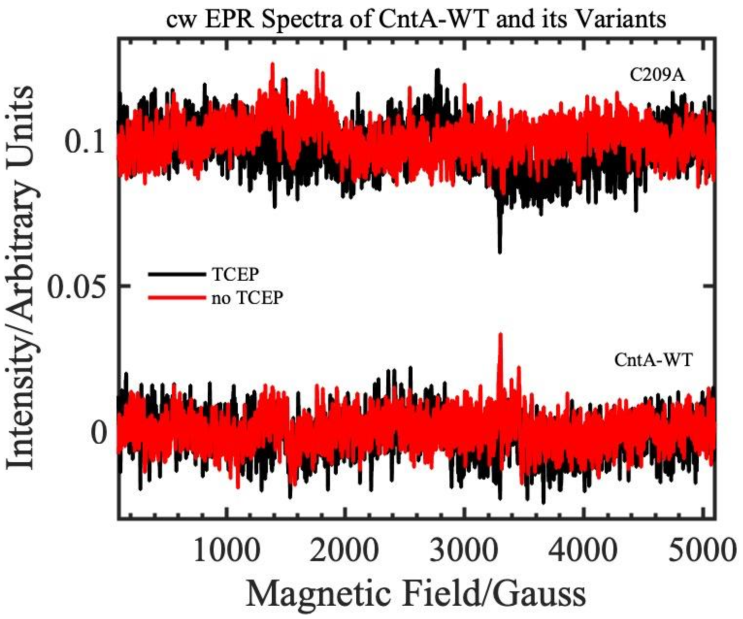

Supplementary Figure 1 cw-EPR spectra of CntA WT and C209A mutant with and without TCEP

Comparisons of cw-EPR spectra of the resting state *Ab*CntA-WT and single mutant, *Ab*CntA-C209A in the presence (black traces) and absence of TCEP (red traces). The spectra were measured as a frozen solution at 20 K. *Conditions* – as in **Figure 4B**.

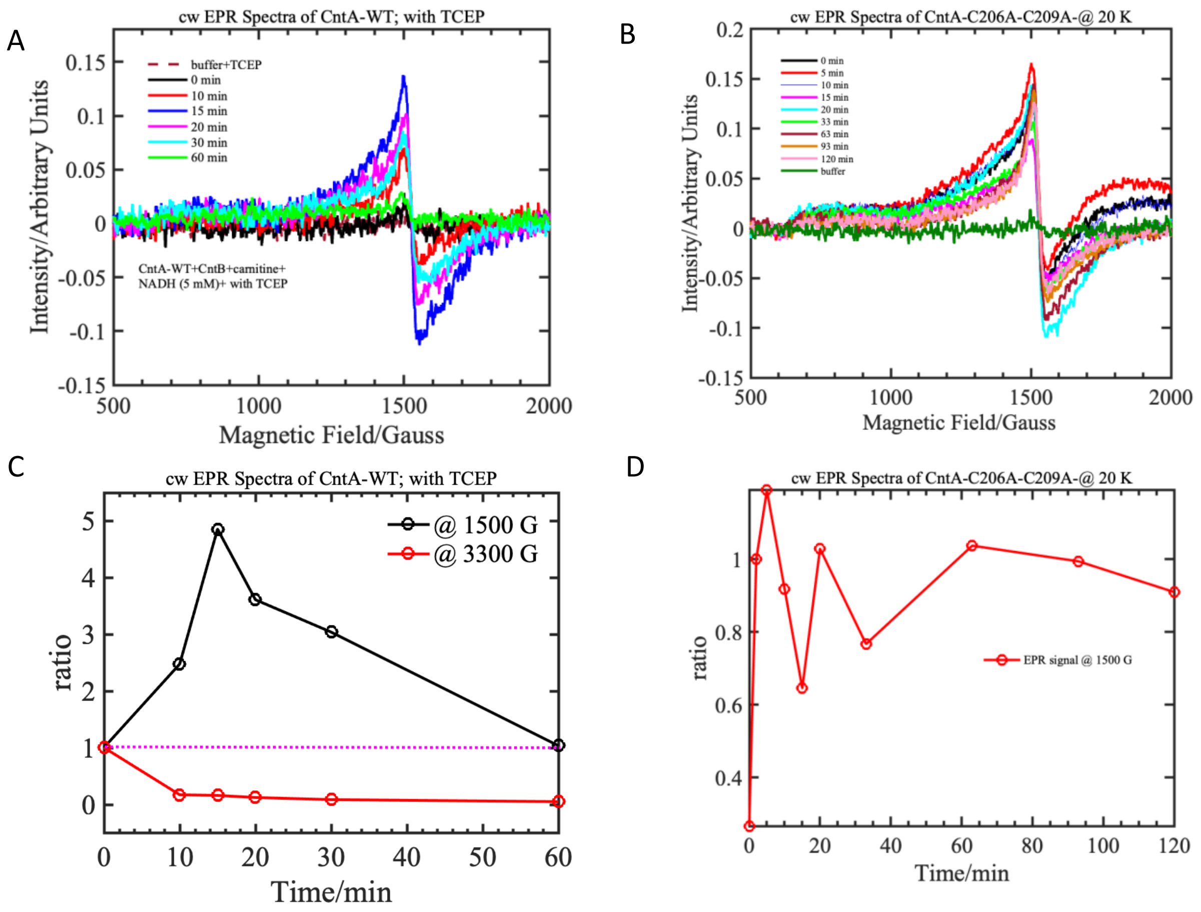

Supplementary Figure 2. **Key figures from main manuscript to illustrate off pathway oxidation**

Comparisons of cw-EPR spectra of [AbCntA-WT+CntB+NADH+carnitine] and [AbCntA-C206A-C209A+CntB+NADH+carnitine] *in the presence of TCEP in the buffer***,** *measured as a frozen solution at 20 K. These sample were annealed/thawed at room temperature for the specified duration mentioned in the figure legend and measured again at 20 K (panels A and B). The observed changes in the spectrum are monitored as a function of annealing (****Figure 8****) time (panels C and D).* These comparisons show the key differences in the observed high-spin, ferric EPR signals of CntA-WT and CntA-C206A-C209A samples in the presence of TCEP when annealed at room temperature for longer time; Panel A – Similar to Figure 7C but in the presence of TCEP in the buffer, Panel B – Figure 9B. Panel C – Figure 8, top left. Panel D – Figure 9 C.

Supplementary Table 1: Estimated CD secondary structure content

	CntA Protein variant							
**Secondary structure Content %**	WT	E205A	C206A	C209A	C206A C209A	C206S	C209S	C206 SC209S
Helix	3.8	3.7	5.2	4.8	5.8	4.3	6.6	3.9
Antiparallel	18	20.4	17.2	20	12.5	19.9	11	31.3
Parallel	8.7	7.5	9.7	8.3	11.8	7.1	11.6	0.5
Turn	21	20.5	21.1	19.6	22.1	21	22.1	16.3
Others	48.5	47.9	46.8	47.3	47.9	47.8	48.6	47.9
Total	100	100	100	100	100.1	100.1	99.9	99.9

Supplementary Table 2: Thermal Shift assay summary of temperature shifts relative to CntA WT.

CntA Protein	Tm (°C)	± SD	ΔTm vs WT
WT	52.67	0.29	-
C206A	51.25	0.65	-1.42
C209A	52.75	0.50	+0.08
C206A/C209A	52.50	0.00	-0.17
C206S	53.13	0.25	+0.46
C209S	51.88	0.25	-0.79
C206S/C209S	53.17	0.29	+0.50

Supplementary Table 3: Crystallographic statistics

C209A+Carnitine (PDB Code: 6Y9C)			
**Wavelength**		**CC(work)**	0.462 (0.305)
**Resolution range**	31.55 - 1.8 (1.864 - 1.8)	**CC(free)**	0.531 (0.283)
**Space group**	P 63	**Number of non-hydrogen atoms**	3119
**Unit cell**	91.2422 91.2422 87.3661 90 90 120	**macromolecules**	2967
**Total reflections**	450475 (31644)	**ligands**	19
**Unique reflections**	38267 (3804)	**solvent**	133
**Multiplicity**	11.8 (8.3)	**Protein residues**	369
**Completeness (%)**	94.62 (99.63)	**RMS(bonds)**	0.007
**Mean I/sigma(I)**	4.15 (0.36)	**RMS(angles)**	1.22
**Wilson B-factor**	18.82	**Ramachandran favored (%)**	93.92
**R-merge**	0.1739 (1.35)	**Ramachandran allowed (%)**	5.52
**R-meas**	0.1816 (1.442)	**Ramachandran outliers (%)**	0.55
**R-pim**	0.05149 (0.4991)	**Rotamer outliers (%)**	0.00
**CC1/2**	0.988 (0.879)	**Clashscore**	14.97
**CC***	0.997 (0.967)	**Average B-factor**	40.35
**Reflections used in refinement**	36232 (3790)	**macromolecules**	40.63
**Reflections used for R-free**	1102 (113)	**ligands**	38.77
**R-work**	0.2483 (0.3740)	**solvent**	34.34
**R-free**	0.2923 (0.3695)	**Number of TLS groups**	3
